# Supplementary material for: Plasma Concentrations of Matrilysins (MMP-7, MMP-26) and Stromelysins (MMP-3, MMP-10) as Diagnostic Biomarkers in High-Grade Serous Ovarian Cancer Patients
Source: Int J Mol Sci. 2025 Jun 13;26(12):5661. doi: 10.3390/ijms26125661 (PMC12193035; doi:10.3390/ijms26125661)
Supplement: Supplementary file 1 [file ijms-26-05661-s001.zip › ijms-3646369-supplementary.pdf]

1. χ^2 test
    1.1. **Table S1.** χ^2 test between oncological status (Ovarian cancer patients and healthy women) and menopausal status

	Premenopausal	Postmenopausal	*Total*	p
**Cancer**	22 (22.0%)	78 (78.0%)	100	0.00113
**Healthy**	24 (48.0%)	26 (52.0%)	50	
Total	46 (30.7%)	104 (69.3%)	**150**	

According to the value of the χ^2 test, there is a statistically significant association (p = 0.00113) between menopausal status and oncological status (absence or presence of cancer) at the significance level of α = 0.05.

1.2. **Table S2.** χ^2 test between oncological status (Ovarian cancer patients and begin lesion patients) and menopausal status

	Premenopausal	Postmenopausal	*Total*	p
**Cancer**	22 (22.0%)	78 (78.0%)	100	0.00009
**Benign**	37 (50.7%)	36 (49.3%)	73	
Total	59 (34.1%)	114 (65.9%)	**173**	

According to the value of the χ^2 test, there is a statistically significant association (p = 0.00009) between menopausal status and oncological status (presence of a benign lesion or presence of cancer) at the significance level of α = 0.05.

1.3 **Table S3.** χ^2 test between oncological status (Ovarian cancer patients and begin lesion patients + healthy women) and menopausal status

	Premenopausal	Postmenopausal	*Total*	p
**Cancer**	22 (22.0%)	78 (78.0%)	100	0.00002
**Benign+Healthy**	61 (49.6%)	62 (50.4%)	123	
Total	83 (37.2%)	140 (62.8%)	**223**	

Based on the χ^2 test results, a statistically significant association was observed (p = 0.00009) between menopausal status and oncological status (defined as the presence of a benign lesion combined with absence of malignancy, or the presence of malignancy), at a significance level of α = 0.05.

2. Evaluation of the Diagnostic Power of Tests by ROC Function for MMPs + CA125 combined analysis
    2.1. ROC curve analysis for the studied parameters in serous OC total group

Table S4. ROC curve analysis for the studied parameters in serous OC total group

Parameter	**AUC**	**SE**	**95% C.I.(AUC)**	**p (AUC=0.5)**

**MMP-3 + CA125**	0.9840	0.0066	(0.971-0.997)	**0.0000**
**MMP-7 + CA125**	0.9993	0.0007	(0.998-1.001)	**0.0000**
**MMP-10 + CA-125**	0.9912	0.0046	(0.982-1.000)	**0.0000**
**MMP-11 + CA-125**	0.9860	0.0059	(0.974-0.998)	**0.0000**
**MMP-26 + CA-125**	0.9901	0.0048	(0.981-1.000)	**0.0000**

Performing combined analyses resulted in an increase in AUC values. The highest AUC was observed for the combined analysis of MMP-7 and CA-125 (AUC = 0.9993), with a similarly high AUC obtained for the combination of MMP-10 and CA-125 (AUC = 0.9912).

2.2. ROC curve analysis for the studied parameters in serous OC I-II stage.

Table S5. ROC curve analysis for the studied parameters in serous OC I-II stage.

Parameter	**AUC**	**SE**	**95% C.I.(AUC)**	**p (AUC=0.5)**
**MMP-3 + CA125**	0.9571	0.0189	(0.920-0.994)	**0.0000**
**MMP-7 + CA125**	0.9976	0.0023	(0.993-1.002)	**0.0000**
**MMP-10 + CA-125**	0.9740	0.0144	(0.946-1.002)	**0.0000**
**MMP-11 + CA-125**	0.9624	0.0172	(0.929-0.996)	**0.0000**
**MMP-26 + CA-125**	0.9757	0.0138	(0.949-1.003)	**0.0000**

In the Ovarian Cancer Stage I–II group, an increase in AUC values was also observed following combined analyses. The highest AUC was recorded for the combination of MMP-7 and CA-125 (AUC = 0.9976).

2.3. ROC curve analysis for the studied parameters in serous OC III-IV stage.

Table S6. ROC curve analysis for the studied parameters in serous OC III-IV stage.

Parameter	**AUC**	**SE**	**95% C.I.(AUC)**	**p (AUC=0.5)**
**MMP-3 + CA125**	0.9955	0.0031	(0.989-1.002)	**0.0000**
**MMP-7 + CA125**	0.9998	0.0001	(0.998-1.004)	**0.0000**

**MMP-10 + CA-125**	0.9986	0.0015	(0.996-1.002)	**0.0000**
**MMP-11 + CA-125**	0.9961	0.0027	(0.991-1.001)	**0.0000**
**MMP-26 + CA-125**	0.9963	0.0025	(0.991-1.001)	**0.0000**

Analyses performed for the OC Stage III–IV group also demonstrated an increase in AUC values. Once again, the highest increase was observed for the combined analysis of MMP-7 and CA-125 (AUC = 0.9998).
